# An attentional limbo: Saccades become momentarily non-selective in between saliency-driven and relevance-driven selection

**DOI:** 10.3758/s13423-022-02091-3

**Published:** 2022-04-04

**Authors:** Elle van Heusden, Wieske van Zoest, Mieke Donk, Christian N. L. Olivers

**Affiliations:** 1 Department of Experimental and Applied Psychology, Faculty of Behavioural and Movement Sciences, Vrije Universiteit Amsterdam, Van der Boechorststraat 7, 1081BT Amsterdam, Netherlands; 2 School of Psychology, University of Birmingham, Hills Building 2.20 Edgbaston, Birmingham, B15 2TT UK

**Keywords:** Saliency-driven selection, Goal-driven selection, Eye movements, Priority map, Visual search

## Abstract

Human vision involves selectively directing the eyes to potential objects of interest. According to most prominent theories, selection is the quantal outcome of an ongoing competition between saliency-driven signals on the one hand, and relevance-driven signals on the other, with both types of signals continuously and concurrently projecting onto a common priority map. Here, we challenge this view. We asked participants to make a speeded eye movement towards a target orientation, which was presented together with a non-target of opposing tilt. In addition to the difference in relevance, the target and non-target also differed in saliency, with the target being either more or less salient than the non-target. We demonstrate that saliency- and relevance-driven eye movements have highly idiosyncratic temporal profiles, with saliency-driven eye movements occurring rapidly after display onset while relevance-driven eye movements occur only later. Remarkably, these types of eye movements can be fully separated in time: We find that around 250 ms after display onset, eye movements are no longer driven by saliency differences between potential targets, but also not yet driven by relevance information, resulting in a period of non-selectivity, which we refer to as the attentional limbo. Binomial modeling further confirmed that visual selection is not necessarily the outcome of a direct battle between saliency- and relevance-driven signals. Instead, selection reflects the dynamic changes in the underlying saliency- and relevance-driven processes themselves, and the time at which an action is initiated then determines which of the two will emerge as the driving force of behavior.

**Supplementary Information:**

The online version contains supplementary material available at 10.3758/s13423-022-02091-3.

## Introduction

Decades of research on visual search behavior has been dedicated to the question of how the visual system determines where to look next when trying to find relevant information. The answer often relies on the concept of a *priority map*, a neural representation which ranks locations in space according to their attentional priority. Priority then depends, among other factors (Awh et al., 2012) on two major, simultaneously present forces: First, it is influenced by *saliency,* or local feature contrast (e.g. Itti & Koch, 2000; Theeuwes, 1992, 1994; Treisman & Gelade, 1980; Yantis & Jonides, 1984). Second, priority is influenced by *relevance*, or the extent to which stimulus features correspond to the observer’s control settings, as shaped by task-goals and motivations (e.g. Folk et al., 1992; Peelen & Kastner, 2011; Reeder et al., 2015). Experimentally these driving forces can be directly juxtaposed, and theories regard selection behavior as the quantal outcome of whichever one of these processes dominates the competition for priority, given a particular context of stimulus properties and attentional control settings. As a result, much of the literature has revolved around which stimulus/task combinations may or may not lead to a particular outcome—that is, either saliency- or relevance-driven selection, which has led to rather dichotomous perspectives (see Luck et al., 2021, and the ensuing commentaties, in particular Anderson, 2021, for the most recent expression of this).

A distinct alternative view is that both these processes may dominate within the same stimulus/task context, but they do so at different moments in time. Under this perspective, the priority map becomes a continuously changing landscape in which saliency and relevance information need not necessarily compete for priority simultaneously but follow different dynamics. Curiously, in most current models of visual search, time is not a critical component, and priority settings are assumed to remain effectively unchanged until an object has been selected (only after which it may then become suppressed; Fecteau & Munoz, 2006; Itti & Koch, 2000; Luck et al., 2021; Schade & Meinecke, 2011; Wolfe, 1994; Wolfe et al., 1989). Moreover, the limited temporal resolution of dependent measures such as manual response times (RTs) or modulations of temporally fixed components such as the N2pc in the electroencephalogram (EEG), has meant that models have relied on snapshots of selection. The evidence so far indicates that their dynamics indeed differ, in particular during the time period leading up to the first eye movement. Specifically, studies using simple, well-controlled stimuli have shown that saliency information is rapidly available but short-lived, while relevance information emerges later, and in a more sustained manner (Anderson et al., 2015, 2016; Hunt et al., 2007; Schütt et al., 2019; Van Zoest et al., 2004; Van Zoest & Donk, 2008) . Furthermore, studies of natural-scene viewing have shown similar effects, when these consider the first eye movement into a scene (Anderson et al., 2015; Anderson et al., 2016; Anderson & Donk, 2017; Parkhurst et al., 2002; but see Peacock et al., 2019a, 2019b). Consequently, which driving force is reflected in behaviour will depend on when the action is triggered, rather than on the stimulus or the control state a priori. When an action is initiated early, it is likely to be saliency-driven; when triggered late it becomes more likely relevance-driven.

**Fig. 1:**
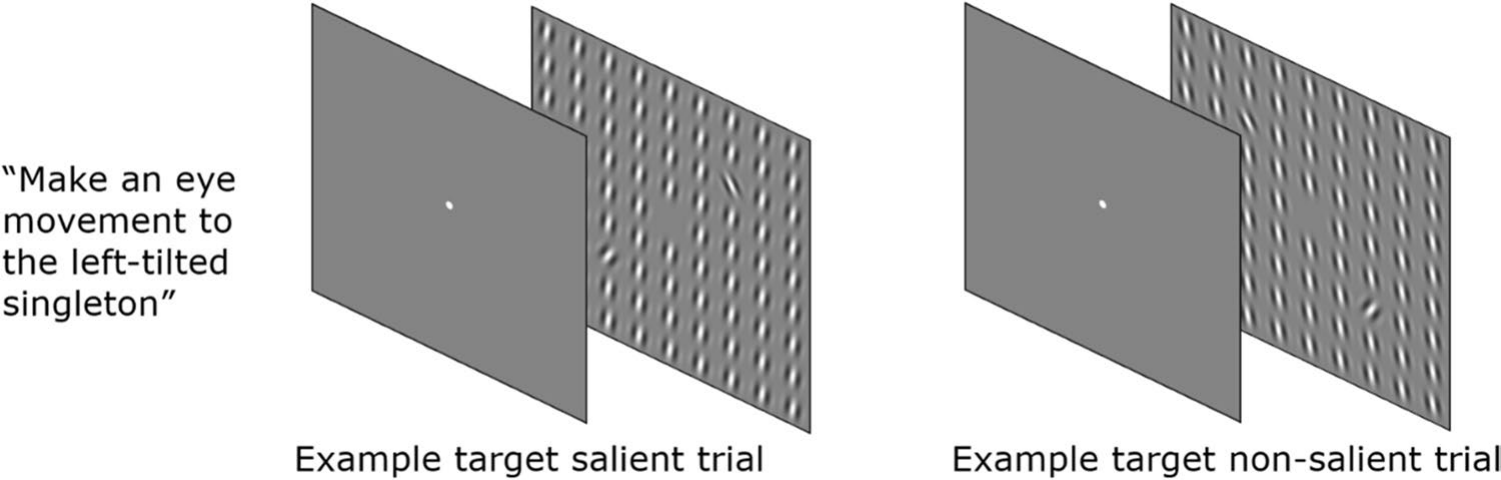
Examples of the two types of trials in Experiment 1. Participants were instructed to make an eye movement to a target singleton (left-tilted for half the number of participants, right-tilted for the other half). Depending on the orientation of the background elements, the target could be more salient than the nontarget (target salient, left display) or less salient (target non-salient, right display)

In support of a dynamic model, here we demonstrate a new phenomenon which shows that saliency- and relevance-driven selection can be completely separated in time, leaving a temporary void in selection. We used a straightforward selection task in which participants were asked to make an eye movement to a relevant target singleton (Fig. 1), which was presented together with an irrelevant nontarget singleton. Depending on the orientation of the background stimuli, the target could be either the most or the least salient of the two singletons. Combined with a recently developed time-resolved eye tracking analysis (van Leeuwen et al., 2019), this task allows for a separation of saliency- and relevance-driven selection across time.

## Methods

Experiments 1 and 2 followed after re-analyses of two existing data sets (see Supplementary Material Figs. S1 and S2) which revealed initial evidence for the existence of a period of almost complete non-selectivity in between saliency-driven and relevance-driven selection, and which we considered as to-be-confirmed pilot data. Here we slightly adapted and repeated the experiments with adequate sample sizes.

### Experiment 1

#### Participants

Twenty participants were recruited (age range 19–25 years, 18 females). All participants reported normal or corrected-to-normal vision and provided written informed consent prior to participation. Participants received either a monetary reward or course credit for taking part in the experiment. The experiment was approved by the ethics review board of the Faculty of Behavioral and Movement Sciences of the Vrije Universiteit Amsterdam and was conducted according to the principles of the Declaration of Helsinki.

#### Apparatus and stimuli

Stimuli were presented on a CRT monitor (1680  × 1050 pixels, 75 Hz). Eye movements were recorded using an EyeLink 1000 Plus eye tracker (SR research, Ontario, Canada). The viewing distance to the screen was fixed at 67 cm by means of a chinrest. Whenever participants were required to fixate, a fixation cross was presented. This fixation cross consisted of two lines (both 0.07 degrees visual angle [dva] in width, and 0.3 dva in height). The search display consisted of 361 Gabor gratings each of which had a diameter of 2 dva and a spatial frequency of 1.5 cycles per degree of visual angle. The Gabors were arranged in a square grid of 19 × 19 elements (30.5 dva × 30.5 dva). The horizontal and vertical distance between the Gabors was 1.7 dva. Search displays consisted of 359 homogeneously oriented background Gabors (all tilted 10° to the left or 10° to the right) and two singleton Gabors, one of which was always tilted 30° to the left and the other 30° to the right. The two singleton Gabors were presented at 4.8 dva from the center of the display and were always presented on one of the screen diagonals. Participants were instructed to make an eye movement to the target which was the left-tilted singleton for one half of the participants and the right-titled singleton for the other half of the participants. Dependent on the orientation of the background elements, on a given trial the target singleton was either more salient (target salient trials) or less salient (target non-salient trials) than the nontarget singleton.

#### Design

A within-subject design was used. All the different combinations of conditions occurred equally often and were presented randomly. Participants completed 1200 trials, divided into 24 blocks of 50 trials each. The first block served as practice and was not included in the analysis. Participants received feedback regarding their average saccade latency after each block of trials. A session took approximately 1.5 h.

#### Procedure

Two examples of the search display are presented in Fig. 1. A 9-point calibration was performed before the start of the experiment (average offset validation: 0.51 dva). Participants received the instruction to make an eye movement to either the left- or right-tilted singleton immediately upon the presentation of the search display. Prior to each trial a drift correction was performed, in which participants were instructed to press the space bar as soon as they were looking at a centrally presented dot (average offset: 0.38 dva). This was followed by the presentation of a central fixation cross. After 500 ms, the search display was presented. To encourage participants to make a fast eye movement, the search display was presented without the fixation cross. The search display was removed from the screen 150 ms after the eye landed within 1.44 dva from one of the two singletons, or after 2000 ms when participants failed to land within 1.44 dva from one the two singletons within that time period.

#### Data analysis

We used the same analysis pipeline for all the data reported in this paper. All data were analyzed offline. The start and endpoints of saccades were defined using the velocity-based algorithm described in Nyström and Holmqvist (2010). We calculated saccade latency (time between search display onset and the start of the first eye movement) and landing position of the first saccade for every trial. A saccade was marked as having selected either of the two singletons if it landed less than half the singleton’s eccentricity away from it. To investigate the time course of visual selection, we used a weighted averaging procedure (van Leeuwen et al., 2019) which mitigates potential distortions that can emerge from standard averaging. Single-subject data were smoothed using a moving Gaussian kernel (10 ms in width). Each point in the time-course (in steps of 1 ms) was given a weight in proportion to the number of data points in that subject’s latency distribution. Weighted average performance was calculated using these weights. As such, this method takes into account the relative contribution of each individual participant to each individual timepoint, preventing group estimates to become unreliable. We used paired t-tests corrected for multiple comparisons in a cluster-based permutation testing procedure (1000 permutations) to test for differences between time courses (Maris & Oostenveld, 2007).

We fitted different alternative models to our data (see Results section). The relative goodness of fit of the alternative best-fitting models were compared using the Akaike information criterion (AIC). AIC was computed as follows (Johnson & Omland, 2004):

1 $$AIC=2\left(\frac{n}{2}\ \ln \left(\frac{RSS}{n}\right)\right)+2\mathrm{k},$$

where *n* is the number of data points, *k* the number of free parameters and *RSS* the residual sum of squares. Lower *AIC* values reflect a better fit. The goodness of fit of two alternative models were compared by calculating the relative likelihood (RL) using Eq. 2:

2 $$RL={e}^{\left(\frac{AIC_{min}\hbox{-} {AIC}_i}{2}\right)},$$

where *AIC*_*min*_ is the AIC value of the best-fitting model and *AIC*_*i*_ is the *AIC* value of the alternative model. *RL* then gives the probability that the alternative model (with the higher AIC value) is a better model than the best-fitting model (with the lowest AIC value).

#### Data exclusion

Trials were excluded from further analysis if the first saccade was initiated earlier than 80 ms or later than 500 ms after display onset (7.64%). An additional 23.3% of trials were removed because the saccade was neither directed towards the target nor the nontarget singleton or could not be detected because of data loss. Trials in which the first eye movement did not select either one of the two singletons were removed because we were interested in the relative selection bias of one over the other singleton. Of the remaining dataset, trials in which saccade latency fell within the lowest 2.5% of the overall latency distribution were also removed (see van Heusden et al., 2021).

## Results

### Oculomotor behavior reveals a period of reduced selectivity

**Fig. 2:**
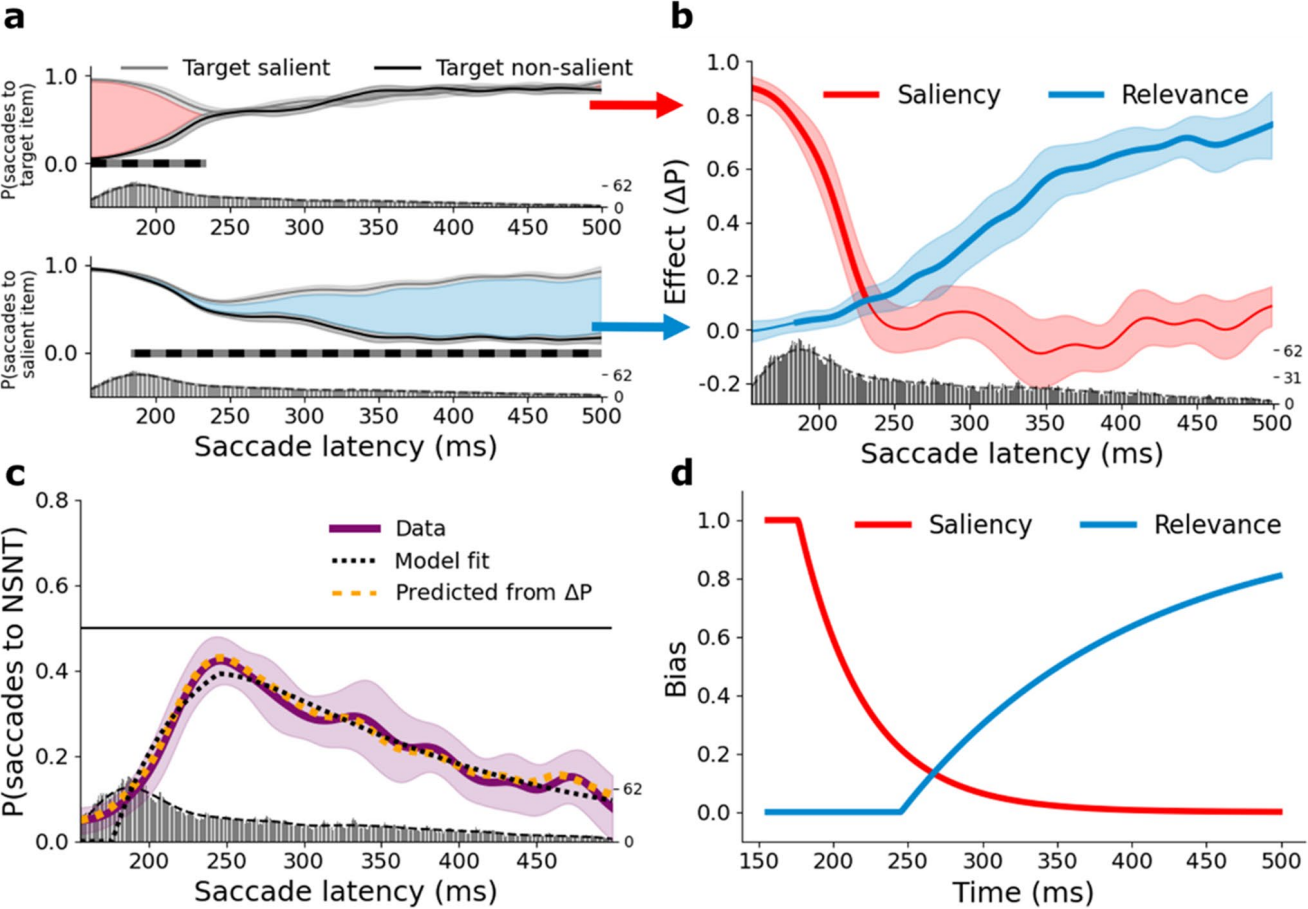
Results of Experiment 1**.** **a** Proportion of trials in which the target (top panel) or the salient item (bottom panel) was selected as a function of saccade latency, plotted separately for target salient and target non-salient trials. Shaded areas correspond to 95% confidence intervals. The clusters of time points at which performance differs between target salient and target non-salient trials are indicated by the grey-black horizontal bars. The red and blue areas reflect the saliency and relevance effect, respectively. The bottom of both subplots shows the saccade latency distribution, including a Kernel Density Estimation (KDE). **b** Difference functions reflecting the net saliency and relevance effects across saccade latency. Shaded areas correspond to 95% confidence intervals. Bold lines indicate where performance is significantly different from zero. The bottom of the plot shows the saccade latency distribution, including a KDE. **c** Proportion of saccades towards the non-salient nontarget (NSNT), as a function of saccade latency. Shaded areas correspond to 95% confidence intervals. The horizontal black line at 0.5 corresponds to purely random selection behavior. Bold lines indicate where the data are different from 0.5. The predicted proportions derived from the full model fit is overlaid in black. The predicted proportions as derived from the observed saliency and relevance effects is overlaid in orange. The saccade latency distribution, including a KDE is shown at the bottom of the plot. **d** The best-fitting functions derived from the full model: S(t) reflecting the probability that selection is biased by saliency and R(t) reflecting the probability that selection is biased by relevance

To investigate the influence of *saliency* as a function of saccade latency, we calculated the proportion of saccades going to the target as a function of saccade latency, separately for target salient (gray) and target non-salient (black) trials (top panel Fig. 2a). As the relative saliency of the target item was the only difference between these trial types, the difference scores provide the net effect of *saliency.* This difference score corresponds to the red shading in Fig. 2a and is shown as the red line in Fig. 2b. To investigate the influence of *relevance* as a function of saccade latency, we calculated the proportion of saccades going to the salient item as a function of saccade latency, separately for target salient (gray) and target non-salient (black) trials (bottom panel, Fig. 2a). As the relative relevance of the salient item was the only difference between these trial types, here the subtraction provides the net effect of *relevance*. The difference score corresponds to the blue shading in Fig. 2a and is shown as the blue line in Fig. 2. Replicating earlier findings (van Heusden et al., 2021; Van Zoest et al., 2004) we find that the effect of saliency is initially strong but rapidly disappears with increasing saccadic latency. The effect of relevance emerges at a later point in time and is more sustained. Crucially, we observe a brief time period in between (starting roughly 240 ms after display onset), during which both effects are approximately zero, implying that selection of an item is neither affected by the relative saliency nor by its relevance during this period of time (see Fig. 2b).

To examine this further, we assessed the specific subset of trials in which a salient target was presented alongside a non-salient nontarget (NSNT). Note that the NSNT is neither the most salient item nor the relevant item in the display, and an ideal system should therefore never select it. Conversely, a period of non-selectivity should come with a momentary increase in NSNT selection. The proportion of trials in which the NSNT was selected as a function of saccade latency is plotted in purple in Fig. 2c. Indeed, saccades made early or late in the trial avoided the NSNT. However, in between, the NSNT was selected on as many as 42.3% of trials, peaking at 246 ms after display onset—where 50% would reflect complete non-selectivity.

Next, we assessed if selection of the NSNT (Fig. 2c) is predicted by the observed data shown in Fig. 2b. If selection performance is indeed entirely determined by the two dynamic components as depicted in Fig. 2b, the probability of selecting the NSNT at time point *t* can be predicted as follows:

3 $$P{(NSNT)}_t=\left(1-S{(t)}_{obs}\right)\ast \left(1-R{(t)}_{obs}\right)\ast 0.5,$$

in which *S*(*t*)_*obs*_ corresponds to the observed net saliency effect, and *R*(*t*)_*obs*_ to the observed net relevance effect as depicted in Fig. 2b. *P*(*NSNT*)_*t*_ is plotted in orange in Fig. 2c. It shows a strong similarity to the observed time course (all clusters, *p* > .94). Note that this similarity is not self-evident, for *P*(*NSNT*) (derived from performance on target salient trials only) is in principle independent of the net saliency and relevance effects (derived from performance differences between target salient and target non-salient trials).

### Reduced selectivity can be best explained by independent time courses

To test whether the momentary non-selectivity indeed stems from two independent dynamic components, we fitted a model (Dombrowe et al., 2012; Donk & van Zoest, 2011; Heimler et al., 2014, 2015). The model incorporates two components: First, a saliency-driven bias, *S*(*t*), reflects the probability that the visual system is biased by saliency, and is defined as:

4 $${\displaystyle \begin{array}{c}S(t)={\mathrm{e}}^{-{a}_S\left(t-{t}_{0S}\right)} for\ t>{t}_{0S},\\ {}S(t)=1\ for\ t\le {t}_{0S},\end{array}}$$

with *t* representing the time since the presentation of a display, *a*_*S*_ reflecting the rate of the function, and *t*_0*S*_ indicating the time since display onset at which the function starts to decrease. Function values range from 1 to 0. Second, a relevance-driven bias, *R*(*t*), reflects the probability that the visual system is subject to a relevance-driven bias and is defined as:

5 $$\begin{array}{c}R(t)=1-\mathrm e^{-a_R\left(t-t_{0R}\right)}\;for\;t>t_{0R},\\R(t)=0\;for\;t\leq t_{0R},\end{array}$$

with *t* representing the time since the presentation of a display, *a*_*R*_ indicating the rate of the function, and *t*_0*R*_ representing the time since display onset at which the function starts to increase. Function values range from 0 to 1. The probability of selecting the NSNT at time point *t* can be predicted as in Eq. 3, but now *R*(*t*) and *S*(*t*) are modeled rather than observed. Given the two components, the model allows for a non-monotonic rise and fall of *P*(*NSNT*)_*t*_ as a function of saccade latency. We contrasted this *full* model with four other models: The *saliency only* and *relevance only* models incorporate either one of the components defined above (*S*(*t*) or *R*(*t*)), but not both, and thus each describe a monotonic relationship using two parameters. The *time-invariant* model has a saliency and a relevance component but assumes the probability that the visual system is biased by either to be constant over time and thus has only one parameter. Note that this model comes closest to existing theories of visual selection in search, which either do not currently incorporate time or assume activation to be constant. Note further that the presence of different time courses per se does not necessarily imply independence, as these could be the result of one signal influencing the other. That is, saliency effects may only be short-lived exactly *because* relevance-driven control mechanisms take over, effectively quashing the influence of a continuous underlying saliency signal on the priority map (e.g. Luck et al., 2021). Or likewise, relevance-based biases may be continuously present from the start, but only emerge late exactly *because* saliency initially dominates. We therefore included the *interdependent* model, which contains both a diminishing saliency component and a rising relevance component, but unlike in the full model, here the decline of *S* is directly coupled to the rise of *R*, such that *t*_0*S*_ = *t*_0*R*_ and *a*_*S*_ = *a*_*R*_ (and thus the model has two parameters). This model thus reflects the case where the different time courses emerge from direct *competition* between saliency- and relevance-driven processes. These models were fitted to the weighted overall group data obtained from target salient trials. Goodness-of-fit was compared using the Akaike Information Criterion (AIC) and pairwise relative likelihood estimates (RL; see Table S1). The analyses showed that of the five models, the full model explained the data best. The estimated time courses of *S*(*t*) and *R*(*t*) derived from the best-fitting full model are plotted in Fig. 2d and yielded the following parameter estimates: *t*_0*S*_ = 176 ms; *a*_*S*_ = 0.022; *t*_0*R*_ = 245 ms; and *a*_*R*_ = 0.007 (see Table S2 for parameters of the other models). Fig. 2c shows the observed *P*(*NSNT*)_*t*_ as a function of saccade latency along with the predicted *P*(*NSNT*)_*t*_ on the basis of the best-fitting full model. The predicted time course of *P*(*NSNT*)_*t*_ reaches a maximum value of 0.391 at 247 ms and corresponds to the maximum in the actual data (0.423 at 246 ms). Note further that the time courses of *S*(*t*) and *R*(*t*) as estimated from *P*(*NSNT*)_*t*_ (Fig. 2d) bear close resemblance to the net empirical saliency and relevance effects shown in Fig. 2b. Finally, to check for any effects of task practice, we compared performance in the first and second half of the experiment (Fig. S3). No differences were found.

Thus, by capitalizing on the temporal variability in saccade generation, we can reveal a brief period during which saccades appear to be in limbo—that is, they are no longer driven by saliency, but also not yet by relevance. To investigate the robustness and generalizability of this observation, we conducted Experiment 2. Here, the task of making an eye movement to a target orientation remained the same, but relative saliency was now defined within an irrelevant dimension, namely color.

### Experiment 2

Experiment 2 represents an earlier collected unpublished dataset that was originally designed for a different purpose. We reverted to this dataset as our labs were closed for prolonged periods of time during the COVID-19 pandemic. Moreover, the experiment followed the same design as the published dataset which here was analyzed as pilot data, see Supplementary Dataset S2.

### Methods

#### Participants

Thirty-two participants recruited at the University of Trento participated in the experiment (age range 19–30 years, 16 females). All participants reported normal or corrected-to-normal vision and gave informed consent prior to participation. Participants received either course credit or a monetary reward for their participation. The Ethics Committee of the University of Trento approved the protocol and this was conducted according to the principles of the Declaration of Helsinki.

#### Apparatus

Stimuli were presented on a 19-in. SVGA color monitor with a resolution of 1024 × 768 pixels resolution and a 75-Hz refresh rate. Eye movements were recorded using and EyeLink 1000 Remote Desktop tracker (SR Research, Ontario, Canada) set to a temporal resolution of 1000 Hz. All participants were tested in a dimly lit room with their heads resting on a chinrest. The monitor was located at eye level, 60 cm from the chin rest.

#### Stimuli and procedure

Displays consisted of one target (i.e., a line oriented 45° to the right), a series of vertical oriented background elements, and one nontarget singleton tilted in the opposite direction of the target (i.e., a line oriented 45° to the left). Elements were presented on a dark gray background, arranged in a 15 × 11 square matrix with a raster width of 30.7° × 25° of visual angle. Elements had an approximate height of 0.9° of visual angle and approximate width of 0.3° visual angle. Targets and nontarget singletons could appear at six different locations. These six potential locations were placed at an imaginary circle in such a way that, embedded in the matrix of nontargets, targets and nontarget singleton were always presented at equal eccentricity from fixation (12.4° of visual angle). When a target and a nontarget singleton were presented, the angular distance between the two elements was always 120°. Stimulus-saliency was manipulated via color and the uniquely colored item was considered the most salient item in the display. In 1/3 of the trials this was the target when it was uniquely colored red, and in 1/3 of trials this was the nontarget singleton when it was uniquely colored green. In the remaining 1/3 of trials none of the elements was uniquely colored making both target and nontarget singleton equally salient. These later trials were not analyzed here. The present design in which the irrelevant color was predictably mapped on to target and distractor was not ideal. One may argue that the color provided another top-down cue, possibly benefiting search for the target. However, this concern was mitigated for several reasons. First, because color was presented unpredictably and was only relevant in 1/3 of the trials, observers were unable to search consistently for color, thus making this strategy not useful. Second and consistent with this, previous work, using a very similar task and stimuli, showed that color mapping did not affect saccadic selection (van Zoest & Donk, 2005). In this work it was found that selection behavior followed the same pattern, irrespective of whether the same or different colors were used for target and distractor (see also Heimler, et al., 2014; Heimler et al., 2015; van Zoest & Donk, 2008 for similar results). Third, if observers would have strategically used the color difference, one would have expected to see a stronger impact of relevance relative to Experiment 1. In other words, top-down control should be available earlier in time, leading to improved performance. As the data will show, this was not the case.

#### Design

A within-subjects design was used. All the different combinations of conditions occurred equally often and presented randomly. Each participant performed 24 practice trials, followed by 432 experimental trials. The three conditions manipulating target and nontarget singleton saliency (colored target, no-colored singletons, colored nontarget singleton) were randomly presented in blocks of trials of 108 trials. Target and nontarget singleton orientations (i.e., right-tilted target with a left-tilted distractor and vice versa) were counterbalanced among participants.

#### Procedure

**Fig. 3:**
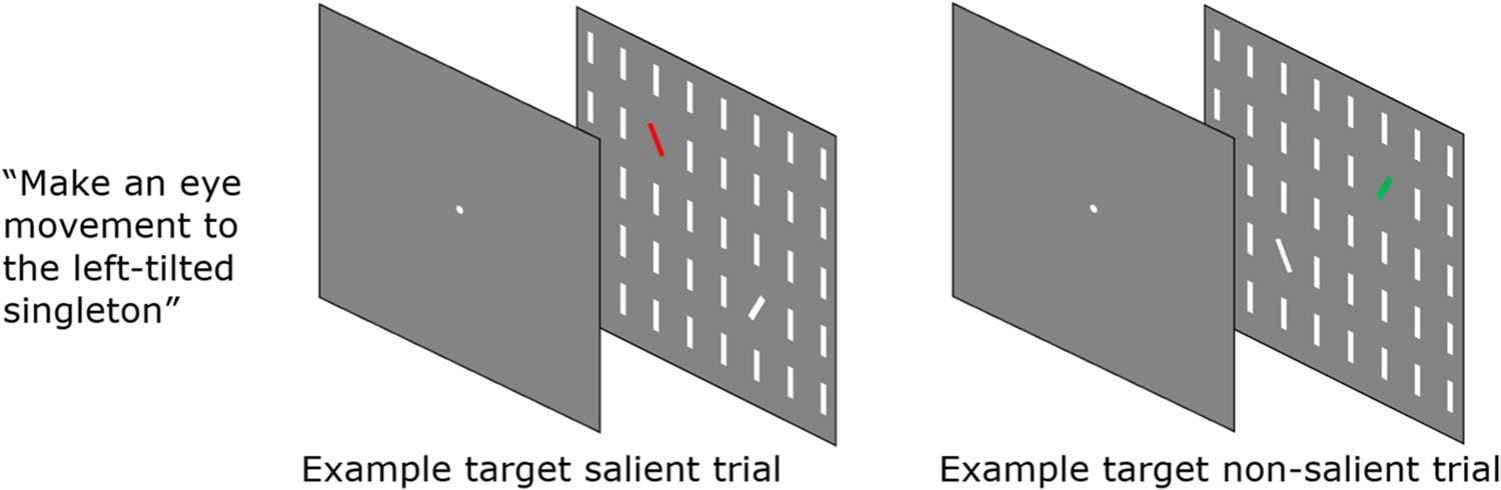
Examples of the two types of trials in Experiment 2. Participants were instructed to make an eye movement to a target singleton (left-tilted for half the number of participants, right-tilted for the other half). Depending on the color of the singletons, the target could be more salient than the nontarget (target salient, left display) or less salient (target non-salient, right display)

Two examples of the search display are presented in Fig. 3. Prior to the recording and every four blocks, the eye tracker was calibrated using the 9-points calibration setup (average offset calibration: 0.45 dva). Participants pressed the space bar to initiate a trial and perform drift correction (average offset: 0.80 dva). A fixation point was then presented for 1000 ms followed by the stimulus matrix for 1500 ms. Participants were instructed to maintain fixation until the search display appeared and then to make a saccadic eye movement to the target as quickly as possible. Feedback on initial saccades mean latencies was provided every 27 trials. The experiment was divided in 16 blocks. Participants were free to take a short break between experimental blocks and a longer break every four blocks, or 108 trials. A session typically took about 50 min.

#### Data exclusion

As in Experiment 1, trials were excluded from further analysis if (1) the first saccade was initiated earlier than 80 ms or later than 500 ms after display onset (0.86%); (2) the saccade was neither directed towards the target nor the nontarget singleton or could not be detected because of data loss (14.9%); or (3) the saccade latency fell within the lowest 2.5% of the overall latency distribution (see van Heusden, Donk, & Olivers, 2021).

## Results

**Fig. 4:**
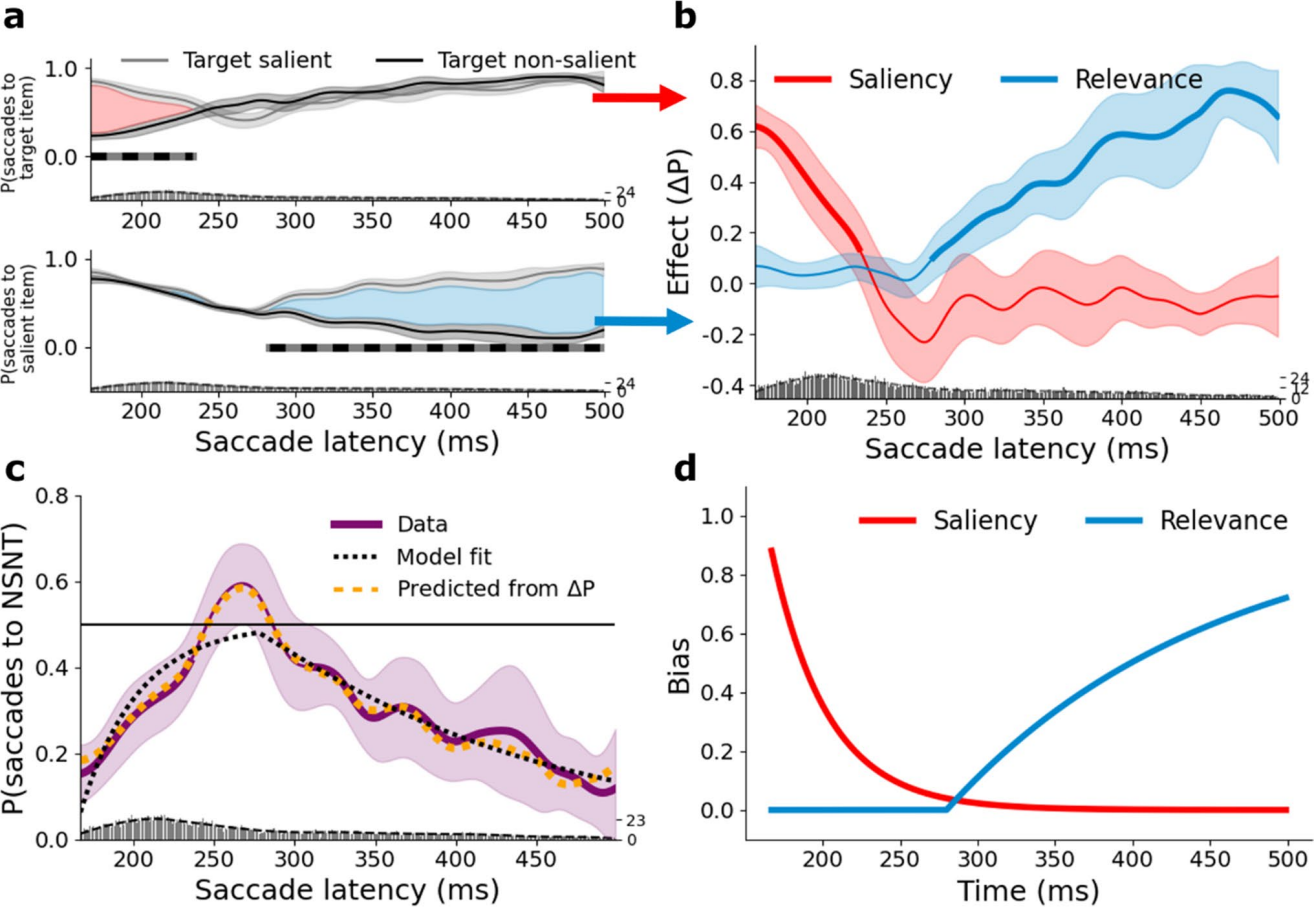
Results of Experiment 2. **a** Proportion of trials in which the target (top panel) or the salient item (bottom panel) was selected as a function of saccade latency, plotted separately for target salient and target non-salient trials. Shaded areas correspond to 95% confidence intervals. The clusters of time points at which performance differs between target salient and target non-salient trials are indicated by the grey-black horizontal bars. The red and blue areas reflect the saliency and relevance effect, respectively. The bottom of both subplots shows the saccade latency distribution, including a Kernel Density Estimation (KDE). **b** Difference functions reflecting the net saliency and relevance effects across saccade latency. Shaded areas correspond to 95% confidence intervals. Bold lines indicate where performance is significantly different from zero. The bottom of the plot shows the saccade latency distribution, including a KDE. **c** Proportion of saccades towards the non-salient nontarget (NSNT), as a function of saccade latency. Shaded areas correspond to 95% confidence intervals. The horizontal black line at 0.5 corresponds to purely random selection behavior. Bold lines indicate where the data is different from 0.5. The predicted proportions derived from the full model fit is overlaid in black. The predicted proportions as derived from the observed saliency and relevance effects is overlaid in orange. The saccade latency distribution, including a KDE is shown at the bottom of the plot. **d** The best-fitting functions derived from the full model: S(t) reflecting the probability that selection is biased by saliency and R(t) reflecting the probability that selection is biased by relevance

As in Experiment 1, the net effects of saliency and relevance were calculated using the proportions of trials in which the target and the salient item were selected respectively (Fig. 4a), revealing a similar set of time courses (Fig. 4b). Here too we observed a brief period in which both the effects of saliency and relevance were low, resulting in observers becoming momentarily non-selective. The pattern of NSNT selection reveals (Fig. 4c) a peak of non-selectivity at 267 ms, where observers selected the NSNT on 59.4% of trials. Moreover, the time course predicted on the basis of the observed differences scores (plotted in orange in Fig. 4c) shows a very strong similarity to the observed time course (all clusters, *p* > .98). Of the five models described previously the full model again explained the data best. The estimates of *S*(*t*) and *R*(*t*) derived from the best-fitting full model are plotted in Fig. 4d, and yielded parameter estimates *t*_0*S*_ = 162; *a*_*S*_ = 0.028; *t*_0*R*_ = 280; *a*_*R*_ = 0.006. The model-predicted *P*(*NSNT*) reached a maximum value of 0.480 at 279 ms (note that the model cannot exceed 0.5). Finally, the estimated time courses of *S*(*t*) and *R*(*t*) in Fig. 4d resemble the net empirical saliency and relevance effects shown in Fig. 4b. Finally, to check for any effects of task practice, we compared performance in the first and second half of the experiment (Fig. S4). No differences were found.

The results of Experiment 2 are very similar to those of Experiment 1, showing that the attentional limbo also occurs when saliency and relevance are defined in different feature dimensions.

## Discussion

Current theories of visual search assume that attentional guidance is the result of saliency and relevance signals projecting concurrently onto a common priority map. Importantly, in these models, signals are as fixed, determined a priori by the stimulus characteristics of the display on the one hand, and the task-induced attentional state of the observer on the other hand. But treating these signals accordingly assumes that they are static. Our data challenges this assumption.

In accordance with earlier work (Donk & Van Zoest, 2008; Van Zoest et al., 2004; Van Zoest & Donk, 2008), we show that eye movements that are triggered early are likely to be saliency driven, while late eye movements are more likely to be relevance driven, despite the target being constant throughout the experiment, allowing for a continuous top-down bias. Importantly, we show that the dynamics of saliency- and relevance-based selection not only differ, but are also completely independent, in that one process can be finished before the other has even started. Empirically, this results in the observation of what we call an *attentional limbo—*a short period of non-selectivity during which saccades are neither affected by the relative saliency of the singletons, nor by their relevance.

We believe the findings help to further resolve a long-standing debate on whether—given a certain combination of stimuli and task—attention during visual search is driven by saliency or relevance (e.g., Carmel & Lamy, 2015; Eimer, 2014; Folk & Remington, 2010; Theeuwes, 2010; Luck et al., 2021). Crucially, this debate has been primarily framed in terms of signal *strength*, rather than signal *dynamics*; it is based on the assumption that both saliency and relevance signals feed into the priority map concurrently, while the strongest signal will then determine selection. Although our account subscribes to the importance of signal strength, we add the important point that this changes over time, and thus whether the stimulus or the task context dominates behavior will be contingent on the moment an action is being triggered. It is noteworthy that models of visual selection often do include a dynamic component, but only *after* an item is being selected. The model described by Itti and Koch (2000), among others, assumes that activation in the saliency map is actively suppressed at locations that were previously visited by the eyes. Similarly, it is believed that when distractors are accidentally attended, attention may rapidly disengage through top-down distractor suppression (e.g. Moher & Egeth, 2012; Theeuwes, 2010). These top-down suppression model predicts that saliency decreases *because* top-down control is taking over. Our modeling approach provides evidence against this type of interdependent model. Instead, oculomotor behavior was best described by a model in which saliency and relevance reflect separate underlying components that act independently, with different time courses, and without direct mutual influence.

**Fig. 5:**
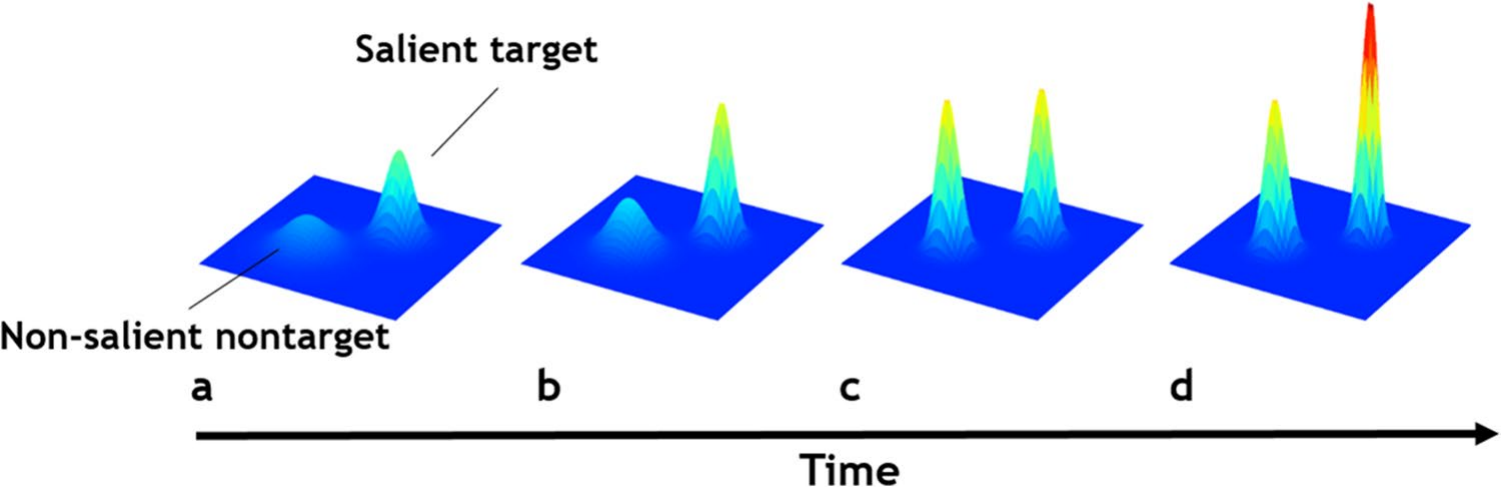
Priority evolving over time. The salient item is processed faster than the non-salient item and therefore generates more activity in the priority map early on (a and b). If a response is triggered during this period of time, the salient item is more likely to be selected, resulting in a saliency effect. In (c), the salient and non-salient items are now both fully segmented from their background and therefore generate about equal levels of activity. If a response is triggered during this period of time, both items are equally likely to be selected, which is expressed as an attentional limbo. Later, in (d), relevance-driven processes further shape the settings in the priority map by e.g., enhancing activity at the location of the relevant item. Responses triggered during and after this time period are driven by relevance. For the salient non-target and non-salient target combination this sequence would look very similar in that the salient non-target initially generates more activity than the non-salient target, until they are both segmented from the background (in c). Later, activity of the non-salient target increases as relevance-driven processes come into play

The idea that the impact of relevance increases over time is understandable from the perspective of a maximally adaptive cognitive system. But why would saliency information rapidly disappear? And why would an adaptive attentional system allow for an episode of apparent non-selectivity? There may be several reasons. First, while a brief availability of saliency information could benefit survival by serving rapid orienting responses, it would be disadvantageous for these signals to continuously affect behavior when they are not also relevant to the organism’s goals. A temporary saliency signal would provide the best of both worlds without the need to invoke additional competitive or inhibitory mechanisms. Second, the cognitive system may eventually be more interested in the *presence* of feature contrasts than in their relative strength. As is illustrated in Fig. 5 the *relative* saliency differences between elements in the display may disappear, while both remain successfully segmented from the background available for relevance-driven selection. Yet stronger feature contrast emerges earlier and initially gains a selection benefit in the priority map. Based on this account, the temporary saliency effects are an emergent property of underlying sensory interactions serving object perception and figure-ground segmentation. We believe this makes sense from the perspective that cognition serves action, and successful interaction with the world is served by knowledge about where objects are in the surrounding, not necessarily by how salient they are. In other words, saliency is ultimately delivering the locations of potential objects of interest for further inspection. Once delivered, the actual saliency of those locations is no longer useful, and subsequent top-down biases are necessary to select the specific target of interest. In this respect, the limbo actually represents an intermediate level of selectivity in which the locations of potential objects are prioritized over other locations in the visual field.

We point out that our results were obtained using relatively simple, well-controlled stimuli and tasks, and may not necessarily speak to visual selection in complex real-world scenes. Overall, eye movements to pictures of real-world scenes have been shown to be predominantly determined by relevance, with if anything only a minor role for saliency (Henderson et al., 2007; Henderson & Hayes, 2017; Peacock et al., 2019a, 2019b). We believe several factors contribute here. First, the heterogeneity of real-world scenes typically also means a heterogeneous landscape of more or less salient locations, rather than the clearly defined signals as used here. Second, real world scenes typically contain gist information (Oliva & Torralba, 2006) indicating where relevant objects may be found. Third, it matters whether one assesses the dynamics of selection within or across eye movements. Analyses of scene perception often involve multiple eye movements across the same scene, and it has been shown that effects of saliency are primarily observed for the first eye movement, and then only if this eye movement is triggered rapidly (Anderson et al., 2015). Later eye movements are predominantly driven by the relevance and/or meaning. In principle our account would predict that a period of apparent non-selectivity should also occur in scene viewing prior to the first eye movement. However, such effects may be quickly superseded by the semantic information that becomes rapidly available for scenes (Kiat et al., 2022; Oliva, 2005). This remains to be investigated in future studies.

In conclusion, the present results emphasize the need to investigate selection as a time-dependent, dynamic process, rather than as the binary outcome of statically defined stimulus and goal states. Rather than a mere aggregation of static bottom-up and top-down inputs, the priority map is highly dynamic, with saliency and relevance signals waxing and waning at different points in time. In extremum this can result in an attentional limbo, providing the first evidence for the complete independence of the activation of saliency- and relevance-based representations.

## Supplementary Information

ESM 1 (DOCX 1420 kb)

## Data Availability

The data are available at OSF (https://osf.io/wfz9j/).

## Declarations

### Open practices

None of the experiments were preregistered. Data and materials can be found at OSF (https://osf.io/wfz9j/).
