# Supplementary material for: An attentional limbo: Saccades become momentarily non-selective in between saliency-driven and relevance-driven selection
Source: Psychon Bull Rev. 2022 Apr 4;29(4):1327–37. doi: 10.3758/s13423-022-02091-3 (PMC8979483; doi:10.3758/s13423-022-02091-3)
Supplement: Supplementary file 1 — (DOCX 1420 kb) [file 13423_2022_2091_MOESM1_ESM.docx]

Supplemental Material

An attentional limbo: saccades become momentarily non-selective in between saliency-driven and relevance-driven selection

**Contents:**

Supplementary Tables S1 and S2

Supplementary Figures S1 – S4

Supplementary Methods

Supplementary tables

Table S1: AIC values for all best-fitting models and the relative likelihoods (RL) calculated in comparison to the lowest AIC value (see main text) separately for the two experiments and the two additional datasets (reported in **Fig. S1** and in **Fig. S2**). Note that the model with the lowest AIC value was always the full model.


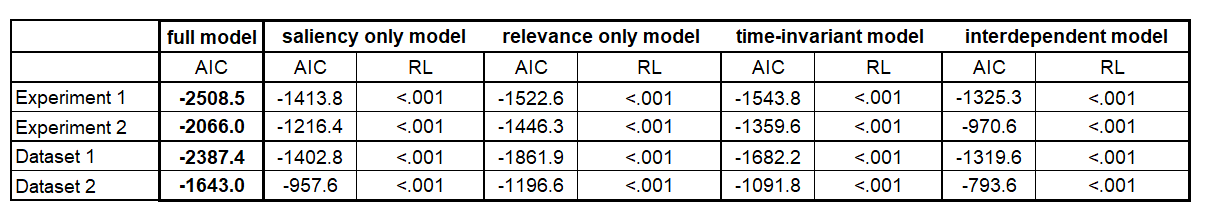


Table S2: parameter estimates derived from the best-fitting full model, saliency only model, relevance only model, time-invariant model, and interdependent model for the two experiments and the two additional datasets. We report group fit parameters obtained by the fit on the grand average data (as shown in Fig.1c, Fig. 2c, Fig. S1d and Fig. S2d).


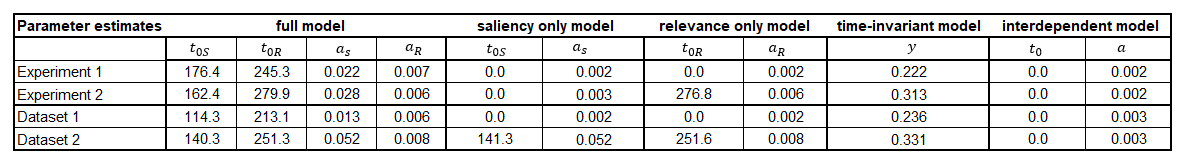


Supplementary figures

Fig. S1.


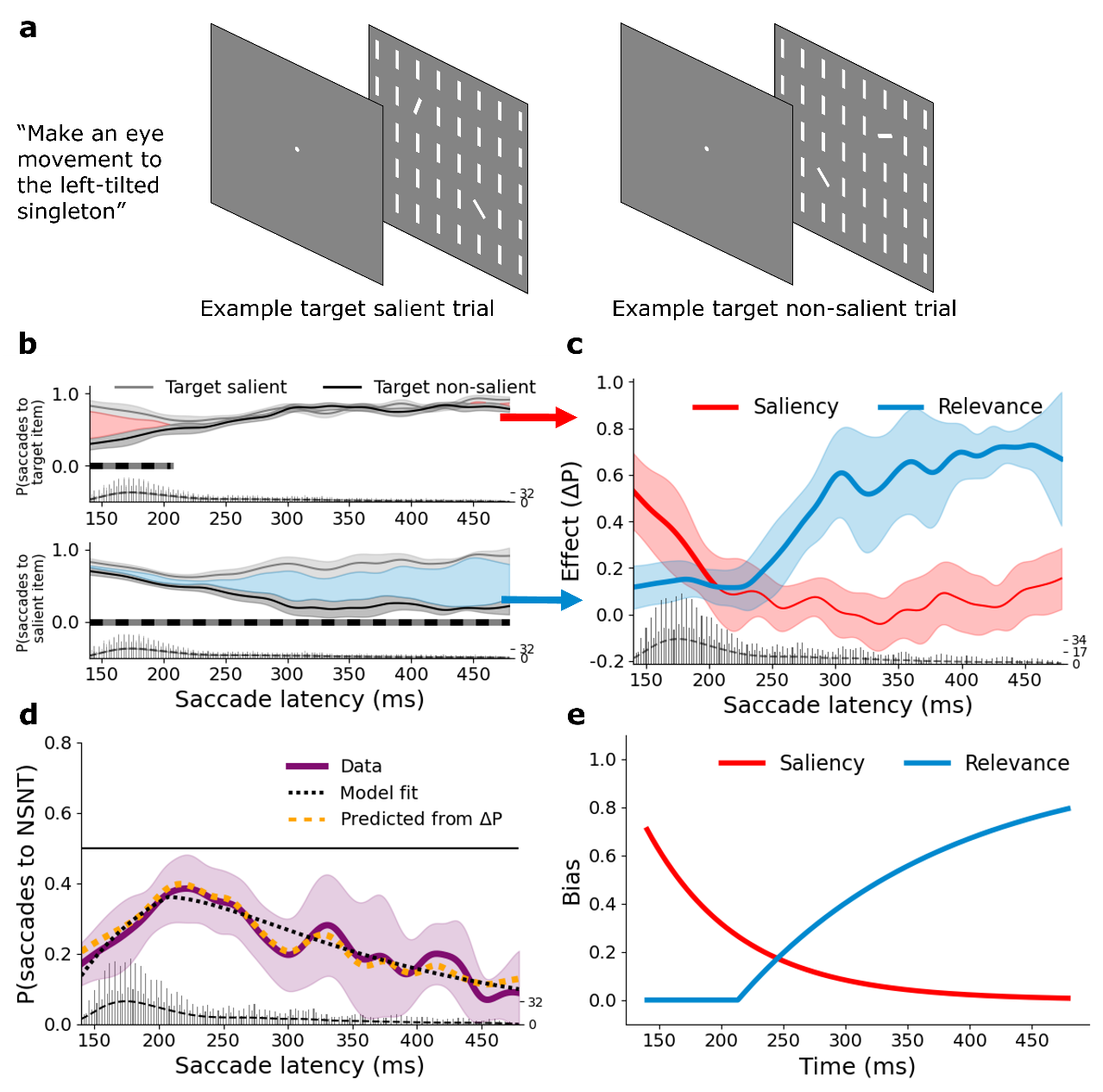


Design and results of experiments in additional dataset 1. (a) Examples of the two types of trials in the experiments corresponding to Additional Dataset 1. Participants were instructed to make an eye-movement to a target singleton (either left-tiled or right-tilted). Depending on the orientation of the nontarget, the target could be more salient than the nontarget (target salient, left display) or less salient (target non-salient, right display). (b) Proportion of trials in which the target (top panel) or the salient item (bottom panel) was selected as a function of saccade latency, plotted separately for target salient and target non-salient trials. Shaded areas correspond to 95% confidence intervals. The clusters of time points at which performance differs between target salient and target non-salient trials are indicated by the grey-black horizontal bars. The red and blue areas reflect the saliency and relevance effect respectively. The bottom of both subplots show the saccade latency distribution, including a Kernel Density Estimation (KDE). (c) Difference functions reflecting the net saliency and relevance effects across saccade latency. Shaded areas correspond to 95% confidence intervals. Bold lines indicate where performance is significantly different from zero. The bottom of the plot shows the saccade latency distribution, including a KDE. (d) Proportion of saccades towards the non-salient nontarget (NSNT), as a function of saccade latency. Shaded areas correspond to 95% confidence intervals. The horizontal black line at 0.5 corresponds to purely random selection behavior. Bold lines indicate where the data is different from 0.5. The predicted proportions derived from the full model fit is overlaid in black. The predicted proportions as derived from the observed saliency and relevance effects is overlaid in orange. The saccade latency distribution, including a KDE is shown at the bottom of the plot. (e) The best-fitting functions derived from the full model: S(t) reflecting the probability that selection is biased by saliency and R(t) reflecting the probability that selection is biased by relevance.

Additional dataset 1 served as the first pilot data set and consists of the data of twenty participants. They were presented with a grid of line segments, two of which were unique singletons: one tilted to the left and one tilted to the right (see **Fig. S1a**). Participants were instructed to make an eye movement to the prespecified target which was either the left-tilted singleton or the right-tilted singleton, and ignore the nontarget, the singleton with the opposite tilt. The relative saliency of target and nontarget was manipulated by varying the tilt of the nontarget singleton, such that either the target or the nontarget had a larger orientation contrast relative to the background than the other. On half of the trials, the target was the most salient singleton (target salient trials), while on the other half the target was the least salient singleton (target non-salient trials). The top panel of **Fig. S1b** shows target selection as a function of saccade latency, plotted separately for target salient and target non-salient trials. By subtracting these two time courses from each other, we were able to assess the net effect of saliency, as the relative saliency of the target item was the only difference between these trial types (**Fig. S1c**). The bottom panel of **Fig. S1b** shows selection of the salient item as a function of saccade latency, plotted separately for target salient and target non-salient trials. By subtracting these two time courses from each other, we were able to assess the net effect of *relevance*, as the relative relevance of the salient item was the only difference between these trial types (**Fig. S1c**). The effect of saliency is initially very strong but rapidly disappears with increasing latency. The effect of relevance is more enduring but only arises gradually at a later point in time. We observed a brief time period in between (starting roughly 200 ms after display onset) where the effects of both saliency and relevance were low. This finding suggests that during this time period, selection was neither driven by saliency nor relevance. To confirm this period of reduced selectivity, we looked into the specific subset of trials in which a salient target was presented alongside a non-salient nontarget (NSNT, **Fig. S1d**). As the NSNT is neither the most salient item nor the relevant item, in an optimal system it should not be selected. If however, there would be a period of non-selectivity in between saliency-driven selectivity and relevance-driven selectivity, then this would be expressed by a momentary increase in NSNT selection, and thus follow a non-monotonic function of saccadic latency. Indeed, as **Fig. S1d** shows, early in the trial observers avoided the NSNT, as here saliency drove the eyes towards the other, more salient, item. Similarly, later in the trial observers avoided the NSNT because relevance drove them to the other, more relevant item, the target. However, in between, at 221 ms after display onset the NSNT was selected on 38.4% of trials. The predicted time course of $P(NSNT)$ on the basis of the observed saliency and relevance effects (plotted in orange in **Fig. S1d**; see main text for further specification) is very similar to the observed time course (all clusters, p > 0.81). To examine how the observed time course of $P(NSNT)$ could be explained best, we fitted the same models as for the main experiments (see main text for model specifics). The analyses showed that overall, the full model explained the data best. The estimated functions of $S(t)$ and $R(t)$ derived from the best-fitting full model are plotted in **Fig. S1e** (with parameter estimates $t_{0S}$ = 114 ms; $a_{S}=0.013$; $t_{0R}$ = 213 ms; $a_{R}=0.006$ ). **Fig. S1d** shows the observed $P(NSNT)$ as a function of saccade latency along with the predicted $P\left( NSNT \right)$ on the basis of the best-fitting full model. The predicted time course of $P\left( NSNT \right)$ reaches a maximum value of 0.363 at 214 ms. Note that the estimated time courses of $S(t)$ and $R(t)$ derived from the best-fitting model (see **Fig. S1e)** bear close resemblance to the net empirical saliency and relevance effects shown in **Fig. S1c.**

Fig. S2.


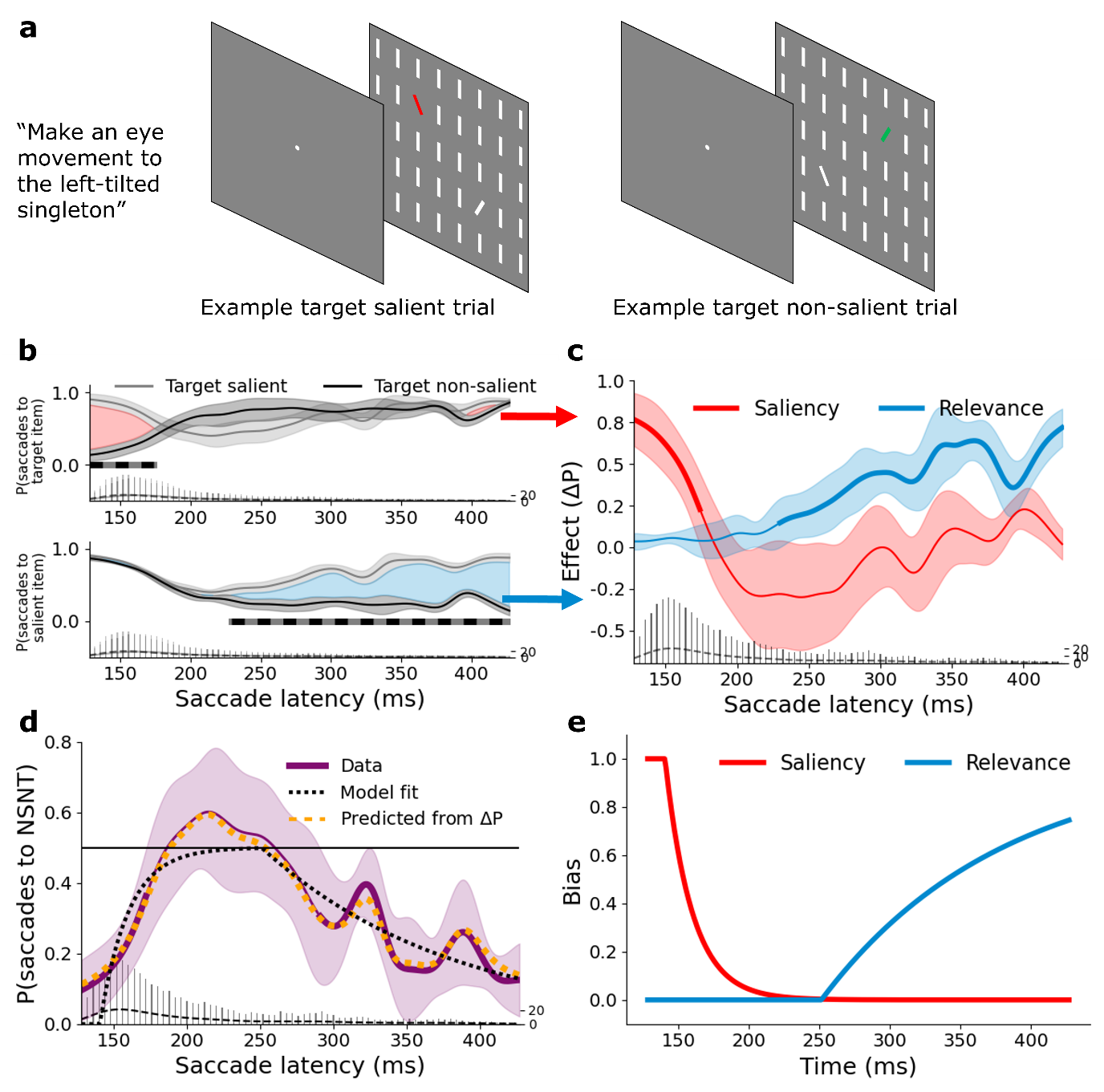


Design and results of experiments in additional dataset 2. (a) Examples of the two types of trials in the experiments corresponding to Additional Dataset 2. Participants were instructed to make an eye-movement to a target singleton. (b) Proportion of trials in which the target (top panel) or the salient item (bottom panel) was selected as a function of saccade latency, plotted separately for target salient and target non-salient trials. Shaded areas correspond to 95% confidence intervals. The clusters of time points at which performance differs between target salient and target non-salient trials are indicated by the grey-black horizontal bars. The red and blue areas reflect the saliency and relevance effect respectively. The bottom of both subplots show the saccade latency distribution, including a Kernel Density Estimation (KDE). (c) Difference functions reflecting the net saliency and relevance effects across saccade latency. Shaded areas correspond to 95% confidence intervals. Bold lines indicate where performance is significantly different from zero. The bottom of the plot shows the saccade latency distribution, including a KDE. (d) Proportion of saccades towards the non-salient nontarget (NSNT), as a function of saccade latency. Shaded areas correspond to 95% confidence intervals. The horizontal black line at 0.5 corresponds to purely random selection behavior. Bold lines indicate where the data is different from 0.5. The predicted proportions derived from the full model fit is overlaid in black. The predicted proportions as derived from the observed saliency and relevance effects is overlaid in orange. The saccade latency distribution, including a KDE is shown at the bottom of the plot. (e) The best-fitting functions derived from the full model: S(t) reflecting the probability that selection is biased by saliency and R(t) reflecting the probability that selection is biased by relevance.

Additional dataset 2 served as the second pilot data set and consists of the data of 12 participants. They were presented with a grid of lines, two of which were unique singletons one tilted to the left and one tilted to the right (see **Fig. S2a**). Participants were asked to make an eye movement to the right-tilted singleton; the left-tilted element was the nontarget singleton). Relative saliency was defined in the color domain, while the two singletons were equally salient in terms of orientation. That is, on target salient trials, the target carried a unique color, while all other stimuli (background elements and nontarget singleton) were presented in white. In contrast, in the target non-salient trials, the nontarget singleton was presented in color. Participants were explicitly told that the color of the items was irrelevant to the task. As for Additional dataset 1, we calculated the net effects of saliency and relevance, which revealed a similar set of time courses (**Fig. S2c**). We observed a brief time period in between (starting roughly 200 ms after display onset) where the effects of both saliency and relevance were low. Here too observers became momentarily non-selective, as the pattern of NSNT selection reveals (**Fig. S2d**). At the peak of non-selectivity, at 215 ms after display onset the NSNT was selected in 60.0% of trials. Again, the predicted time course of $P(NSNT)$ on the basis of the observed saliency and relevance effects (plotted in orange in **Fig. S2d**; see main text for further specification) is very similar to the observed time course (all clusters, p > 0.97). We fitted the same models as for the main experiments (see main text for model specifics). The analyses showed that overall, the full model explained the data best. The estimated functions of $S(t)$ and $R(t)$ derived from the best-fitting full model are plotted in **Fig. S2e** (with parameter estimates $t_{0S}$ = 140 ms; $a_{S}=0.052$; $t_{0R}$ = 251 ms; $a_{R}=0.008$ ). **Fig. S2d** shows the observed $P\left( NSNT \right)$ as a function of saccade latency along with the predicted $P\left( NSNT \right)$ on the basis of the best-fitting full model. The predicted time course of $P\left( NSNT \right)$ reaches a maximum value of 0.5 from 233 to 251 ms.

Fig. S3.


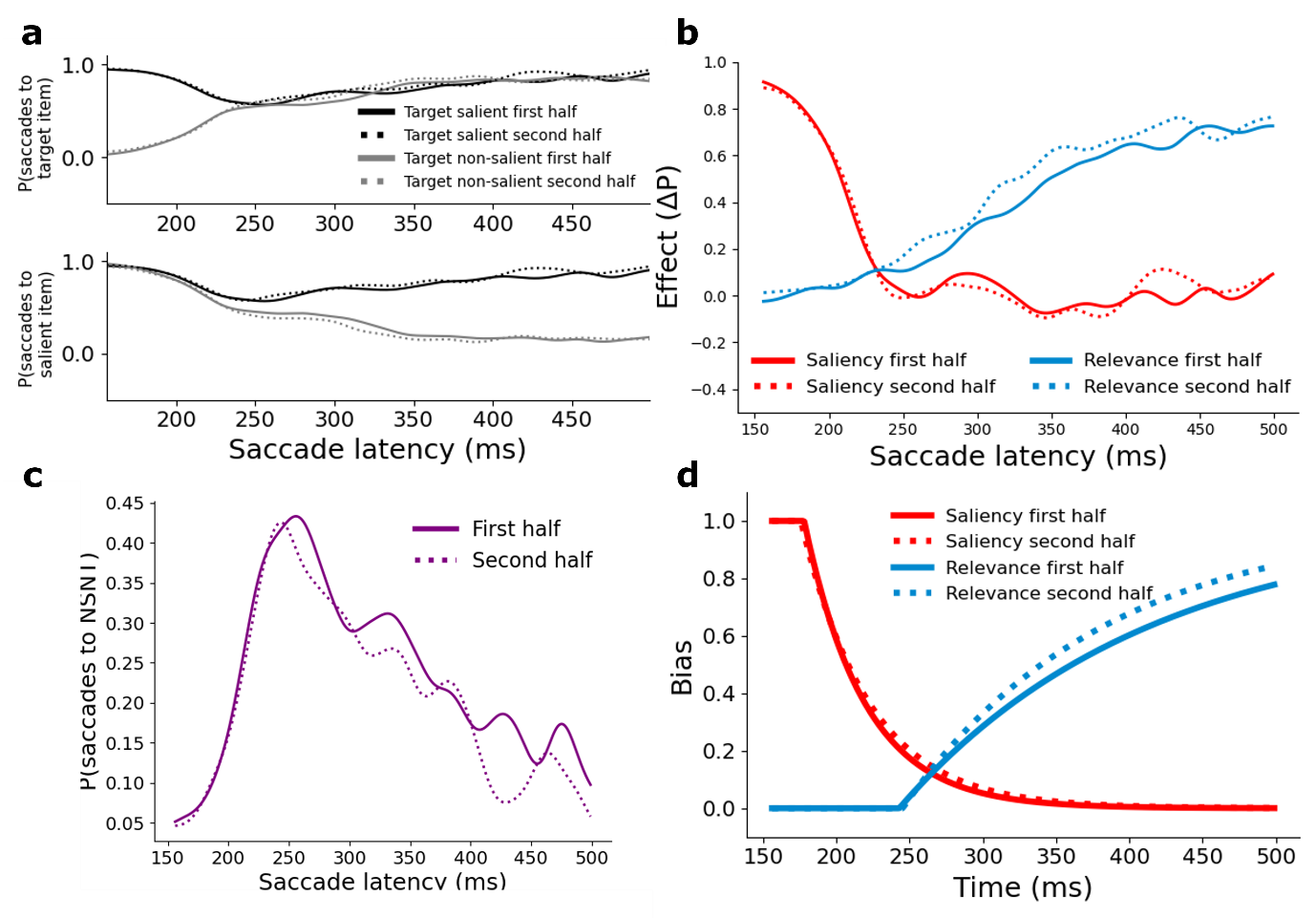


Data split of Experiment 1. (a) Proportion of trials in which the target (top panel) or the salient item (bottom panel) was selected as a function of saccade latency, plotted separately for target salient and target non-salient trials of the first and second half of the experiment. (b) Difference functions reflecting the net saliency and relevance effects across saccade latency, plotted separately for trials from the first and second half of the experiment. (c) Proportion of saccades towards the non-salient nontarget (NSNT) as a function of saccade latency, plotted separately for trials from the first and second half of the experiment. Shaded areas correspond to 95% confidence intervals. (d) The best-fitting functions derived from the full model: S(t) reflecting the probability that selection is biased by saliency and R(t) reflecting the probability that selection is biased by relevance, plotted separately for trials from the first and second half of the experiment.

Fig. S4.


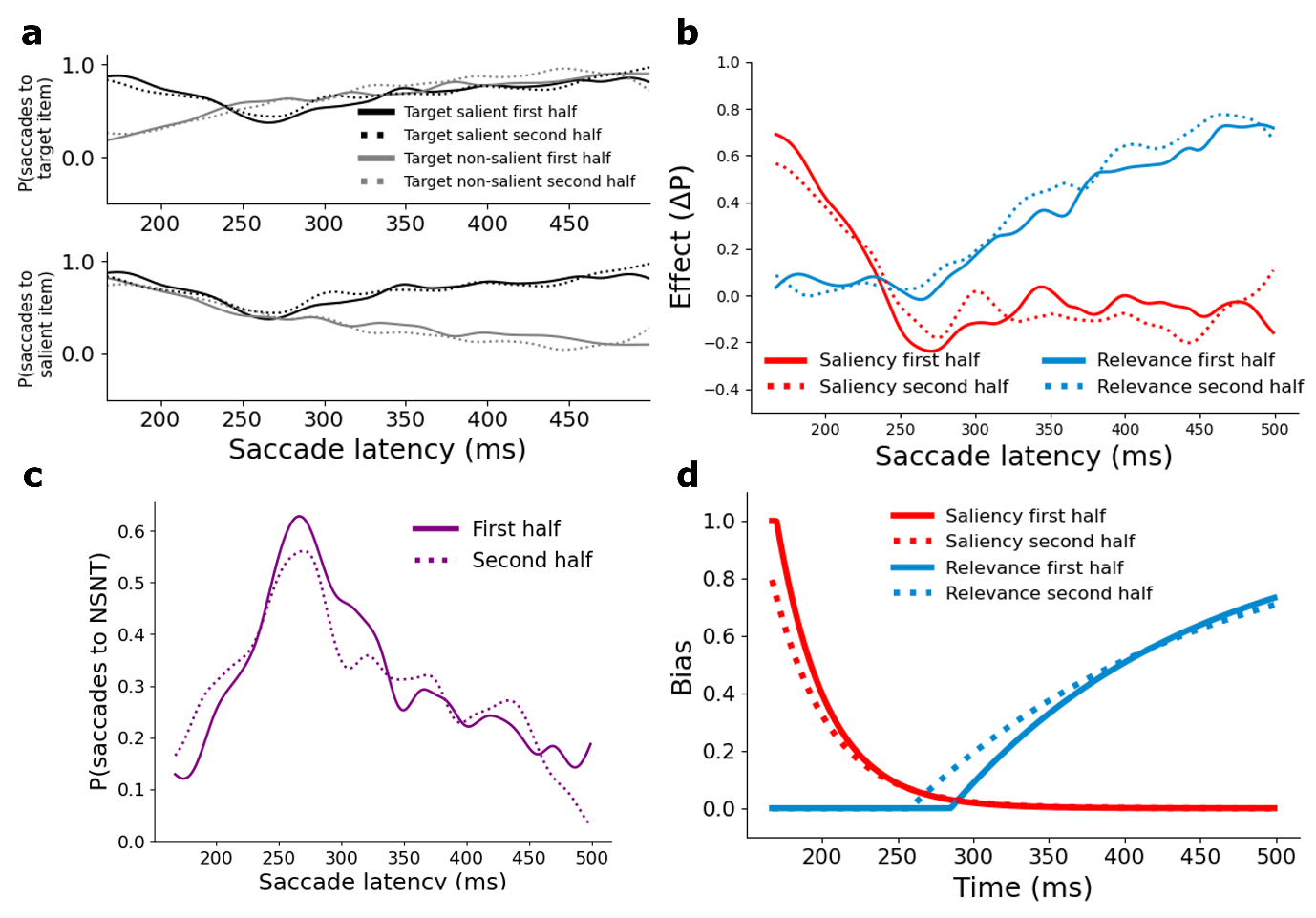


Data split of Experiment 2. (a) Proportion of trials in which the target (top panel) or the salient item (bottom panel) was selected as a function of saccade latency, plotted separately for target salient and target non-salient trials of the first and second half of the experiment. (b) Difference functions reflecting the net saliency and relevance effects across saccade latency, plotted separately for trials from the first and second half of the experiment. (c) Proportion of saccades towards the non-salient nontarget (NSNT) as a function of saccade latency, plotted separately for trials from the first and second half of the experiment. Shaded areas correspond to 95% confidence intervals. (d) The best-fitting functions derived from the full model: S(t) reflecting the probability that selection is biased by saliency and R(t) reflecting the probability that selection is biased by relevance, plotted separately for trials from the first and second half of the experiment.

Supplementary methods

Additional dataset 1

*Data* – We combined the data from two experiments which were previously published. The first dataset (Dataset 1a, N = 12) was published as Experiment 4 in Van Zoest, Donk, & Theeuwes (2004). The second dataset (Dataset 1b, N = 8) was previously published as Experiment 2 in Donk & Van Zoest (2008). The two experiments were very similar (see below) and were therefore combined for the current purpose.

*Apparatus* - Both experiments were performed using the same setup. Stimuli were presented on a SVGA color monitor with a resolution of 1024 x 768 pixels. Distance from the screen was kept constant at 75 cm by the use of a chinrest. Eye movements were recorded using the Eyelink eyetracker (SR research), with a temporal resolution of 250 Hz.

*Stimuli and procedure* – Both experiments used the same stimuli and task, except where indicated. Stimuli were white lines (0.31 x 0.76 dva) presented on a black background. The search display consisted of a grid of vertically oriented lines that contained two singleton elements, each of unique orientation. One of the singletons was a predefined target and had on offset of 45 degrees. The other singleton was the nontarget and could have an offset of either 22.5, 45, or 67.5 degrees (Dataset 1a) or 22.5 or 67.5 degrees (Dataset 1b). The direction of tilt of the nontarget was always opposite to that of the target. All items were presented in a 9 x 13 rectangular grid (17.05 dva x 12.63 dva). Singletons were presented at two of six possible locations at 6.1 dva from fixation. The circular angle difference between target and nontarget was always 120 degrees. Relative target saliency varied in dependency of the tilt of the nontarget – that is, the target was either salient (nontarget: 22.5), non-salient (nontarget: 67.5) or equally salient (nontarget: 45) compared to the nontarget. Trials in which the target and nontarget were equally salient were not analyzed here. Participants pressed spacebar to start a trial, after which a fixation dot was presented for 1000 ms. Then, the search display was presented for 1500 ms. Participants were instructed to look at the target as quickly as possible, and stay fixated on the target until the search display disappeared from screen.

*Design –* In Dataset 1a*,* for half of the subjects the target was right tilted and for the other half the target was left tilted. Relative target saliency (which depended on the tilt of the nontarget) was kept constant within a block of trials. The order of relative target saliency was counterbalanced across participants. The experiment consisted of 36 practice trials and 540 experimental trials. Participants received feedback about their saccade latency after every 30 trials. In Dataset 1b the direction of tilt of the target varied between blocks, such that half of the subjects started with a block in which the target was tilted to the left and then completed a block in which the target was tilted to the right. For the other half of the subjects this was the other way around. Relative target saliency was kept constant within a block of trials, but switched between the two blocks. Here each block consisted of 300 trials and subjects received feedback regarding saccade latency every 25 trials. Before the start of each block, 36 practice trials were conducted.

*Data exclusion -* Trials in which the first saccade was neither directed to the target nor to the nontarget or where a saccade could not be detected because of data loss (11.1%), and those in which the saccade latency fell outside our latency criteria (see Methods main text, 5.8%) were discarded from further analyses.

Additional dataset 2

*Data* - Dataset 2 (N = 12) was previously published as Experiment 1 in van Zoest & Donk (2005).

*Apparatus* – The setup used to collect this dataset was the same as that of Dataset 1.

*Stimuli and procedure* – Stimuli and procedure were the same as for Dataset 1, with the following exceptions: the target always had a 45 degree offset towards the right. The nontarget always had a 45 degree offset to the left. Thus, the two singletons were equally salient in the orientation dimension. However, on 33% of the trials, the target was a red line while both the nontarget and the background elements were white lines (target salient condition). Conversely, on 33% of the trials the nontarget was a red line while the target and background elements were white lines (target non-salient condition). On the remaining 33% of trials, both singletons were white lines. This condition was not further analyzed here. Participants were explicitly told that the color of the items was irrelevant to the task.

*Design –*A within-subjects design was used. The experiment consisted of 48 practice trials and 540 experimental trials, presented in two blocks of 270 trials. The different trial types (target salient, target non-salient, equally salient singletons) were mixed. Participants received feedback about their saccade latency after every 30 trials.

*Data exclusion -* Trials in which the first saccade was neither directed to the target nor to the nontarget or where a saccade could not be detected because of data loss (10.5%), and those in which the saccade latency fell outside our latency criteria (see Methods Main text, 5.9%) were discarded from further analyses.

**References**

Donk, M., & Van Zoest, W. (2008). Effects of salience are short-lived. *Psychological Science*, *19*(7), 733–739. https://doi.org/10.1111/j.1467-9280.2008.02149.x

van Zoest, W., & Donk, M. (2005). The effects of salience on saccadic target selection. *Visual Cognition*, *12*(2), 353–375. https://doi.org/10.1080/13506280444000229

Van Zoest, W., Donk, M., & Theeuwes, J. (2004). The role of stimulus-driven and goal-driven control in saccadic visual selection. *Journal of Experimental Psychology: Human Perception and Performance*, *30*(4), 746–759. https://doi.org/10.1037/0096-1523.30.4.749
